# Expression of Siglec-E Alters the Proteome of Lipopolysaccharide (LPS)-Activated Macrophages but Does Not Affect LPS-Driven Cytokine Production or Toll-Like Receptor 4 Endocytosis

**DOI:** 10.3389/fimmu.2017.01926

**Published:** 2018-01-15

**Authors:** Manjula Nagala, Emma McKenzie, Hannah Richards, Ritu Sharma, Sarah Thomson, Pietro Mastroeni, Paul R. Crocker

**Affiliations:** ^1^Division of Cell Signalling and Immunology, School of Life Sciences, University of Dundee, Dundee, United Kingdom; ^2^Department of Veterinary Medicine, University of Cambridge, Cambridge, United Kingdom

**Keywords:** Siglec-E, toll-like receptor 4, lipopolysaccharide, macrophage, dendritic cells, proteomics

## Abstract

Siglec-E is a murine CD33-related siglec that functions as an inhibitory receptor and is expressed mainly on neutrophils and macrophage populations. Recent studies have suggested that siglec-E is an important negative regulator of lipopolysaccharide (LPS)-toll-like receptor 4 (TLR4) signaling and one report (1) claimed that siglec-E is required for TLR4 endocytosis following uptake of *Escherichia coli* by macrophages and dendritic cells (DCs). Our attempts to reproduce these observations using cells from wild-type (WT) and siglec-E-deficient mice were unsuccessful. We used a variety of assays to determine if siglec-E expressed by different macrophage populations can regulate TLR4 signaling in response to LPS, but found no consistent differences in cytokine secretion *in vitro* and *in vivo*, comparing three different strains of siglec-E-deficient mice with matched WT controls. No evidence was found that the siglec-E deficiency was compensated by expression of siglecs-F and -G, the other murine inhibitory CD33-related siglecs. Quantitative proteomics was used as an unbiased approach and provided additional evidence that siglec-E does not suppress inflammatory TLR4 signaling. Interestingly, proteomics revealed a siglec-E-dependent alteration in macrophage protein composition that could be relevant to functional responses in host defense. In support of this, siglec-E-deficient mice exhibited enhanced growth of *Salmonella enterica* serovar Typhimurium in the liver following intravenous infection, but macrophages lacking siglec-E did not show altered uptake or killing of bacteria *in vitro*. Using various cell types including bone marrow-derived DCs (BMDCs), splenic DCs, and macrophages from WT and siglec-E-deficient mice, we showed that siglec-E is not required for TLR4 endocytosis following *E. coli* uptake or LPS challenge. We failed to see expression of siglec-E by BMDC even after LPS-induced maturation, but confirmed previous studies that splenic DCs express low levels of siglec-E. Taken together, our findings do not support a major role of siglec-E in regulation of TLR4 signaling functions or TLR4 endocytosis in macrophages or DCs. Instead, they reveal that induction of siglec-E by LPS can modulate the phenotype of macrophages, the functional significance of which is currently unclear.

## Introduction

Innate immune cells express toll-like receptors (TLRs) which play critical roles in recognition of various pathogen-associated molecular patterns (PAMPs). Exposure of macrophages and dendritic cells (DCs) to PAMPS, such as Gram-negative bacterial lipopolysaccharide (LPS), which triggers through TLR4, can orchestrate a diverse gene expression program required for shaping the innate and adaptive arms of the immune response (2–4). These changes include the induction or repression of a wide range of genes that regulate pro-inflammatory cytokines, chemokines, inflammatory mediators, polarization, migration, and cell survival. These processes are tightly regulated and loss of control is associated with conditions, such as septic shock and inflammatory diseases (5–7).

Many immune cells express a variety of membrane proteins with cytosolic tyrosine-based inhibitory motifs (ITIMs) that negatively regulate signaling through activation receptors. One important class of such inhibitory receptors implicated in regulation of TLR signaling is the family of siglecs, defined as transmembrane sialic acid-binding Ig-like lectins (8). The CD33-related siglecs are a recently evolved subset that are mainly expressed in a complex manner by cells of the innate immune system. Most contain an ITIM and an ITIM-like motif in their cytoplasmic tails which, following tyrosine phosphorylation by Src-family kinases, are thought to be important for inhibitory signaling *via* recruitment and activation of protein tyrosine phosphatases SHP-1 and SHP-2 (9, 10). The sialic acid-binding sites of inhibitory siglecs on leukocytes are occupied by *cis*-interactions with sialic acids on the plasma membrane [reviewed in Ref. (8)]. Depending on the sialic acid carriers, these *cis*-interactions are likely to be important for regulating the functional responses of siglecs [reviewed in Ref. (11)]. Siglecs can also interact with sialic acid ligands *in trans*, for example, on encountering another cell or a pathogen expressing high-affinity/avidity ligands and this can trigger siglec-dependent signaling functions and endocytosis.

As a model system to understand the signaling functions of inhibitory CD33-related siglecs on myeloid cells, our laboratory has focused on murine siglec-E which is mainly expressed on neutrophils, tissue macrophages, and splenic DCs (12, 13). There have been several reports showing that siglec-E and its human homolog siglec-9 are important for regulation of TLR4-driven cytokine production in macrophages and DCs. In murine bone marrow-derived macrophages (BMDM), cross-linking of siglec-E with antibodies (Abs) reduced the production of TNF-α, IL-6, and RANTES in response to LPS stimulation (14). Overexpression of recombinant human siglec-9 in human THP-1 and mouse RAW264 macrophage cell lines downregulated the production of pro-inflammatory cytokines following LPS stimulation (15). Siglec-E expression has been shown to suppress pro-inflammatory cytokine production by macrophages in response to a sialylated strain of Group B *Streptococcus* (16) and treatment of murine macrophages with sialic acid-decorated nanoparticles was found to abrogate LPS-induced inflammation (17). More recently, Chen et al. reported direct interactions between TLRs and siglecs, including siglec-E (18). The same group also proposed that *cis*-interactions between siglec-E and TLR-4 are required for TLR4 endocytosis following uptake of *Escherichia coli* and are important for downregulating TLR4-mediated inflammatory responses (1, 18).

In this report, we further investigate the potential role of siglec-E in TLR4 signaling *via cis*-interactions using three different lines of WT and siglec-E-deficient mice. Consistent with previous studies, we show that siglec-E is strongly upregulated by low-dose (1 ng/ml) LPS leading to constitutive tyrosine phosphorylation and recruitment of the negative regulator SHP-1. However, we were unable to demonstrate a siglec-E-dependent effect on pro-inflammatory cytokine production by macrophages challenged with a high dose (100 ng/ml) of LPS, using a variety of approaches, including unbiased quantitative proteomics. Furthermore, we failed to see any expression of siglec-E on bone marrow-derived DCs (BMDCs) and were unable to reproduce the previous findings of siglec-E-dependent internalization of TLR4 in response to bacterial challenge. However, we could demonstrate by quantitative proteomics that the phenotype of siglec-E-deficient macrophages challenged with LPS was different from WT macrophages, suggesting that siglec-E contributes to the differentiation of macrophages exposed to LPS, but plays little or no role in directly regulating TLR4-dependent signaling by macrophages or DCs under physiological conditions.

## Materials and Methods

### Materials

Dulbecco’s phosphate-buffered saline (PBS) without Ca and Mg, fetal bovine serum (FBS) (qualified, heat inactivated, E.U.-approved), penicillin and streptomycin solution, Trypsin–EDTA solution, protein G Dynabeads, Microplate BCA Protein Assay, NuPAGE LDS Sample Buffer, NuPAGE^®^ Novex 4–12% Bis-Tris gel, MOPS running buffer, and sample reducing agent, trypsin protease, Pierce MS Grade, TMT 10-plex™ Isobaric Reagent Label Set were from Thermo Fisher Scientific, Paisley, UK; Sera-Mag SpeedBead Carboxylate-Modified Magnetic Particles were from GE LifeSciences; Roche-COMPLETE Mini EDTA-Free Protease Inhibitor tablets, Roche-PHOSS-RO, PhosSTOP™ Trypan blue solution, anti-sheep IgG (whole molecule)-peroxidase and anti-rabbit IgG (whole molecule)–peroxidase Ab produced in goat, lipopolysaccharide from *E. coli* 0111:B4 were from Sigma; GM-CSF and IL-4 were from Peprotech, GolgiStop, CD16/CD32 (Fc block), V500 rat anti-mouse I-A/I-E (clone: M5/114; 562366) were from BD Bioscience, UK; anti-mouse TNF alpha PE (clone: MP6-XT22), anti-mouse CD11c PE-cy7 (Clone: N418), anti-mouse Ly-6G (Gr-1) Alexa Fluor^®^ 488 (clone: RB6-8C5) were from eBioscience, UK; anti-*Salmonella* Typhimurium (clone: 1E6), anti-phosphotyrosine Ab (HRP) (Abcam clone: PY20-ab16389) were from Abcam, UK; APC anti-mouse CD11c Ab (clone: N418), PE-conjugated anti-siglec-E used in flow cytometry (clone: M1304A01), biotin anti-mouse TLR4 (CD284)/MD2 complex Ab (clone: MTS510), PE/Cy7 anti-mouse TLR4 (CD284)/MD2 complex Ab (clone: MTS510), PE anti-mouse/human CD11b Ab (clone: M1/70), APC/Cy7 anti-mouse Ly-6G/Ly-6C (Gr-1) Ab (clone: RB6-8C5) were from Biolegend, UK; and anti-mouse SHP-1 Ab (clone: C-19) was from Santa Cruz. *E. coli* 0111:B4 LPS (Sigma) was used in all *in vitro* experiments. *E. coli*-GFP was obtained from the American Type Culture Collection (25922GFP). Affinity purified sheep anti-siglec-E Ab was produced in-house (12) and used for immunoprecipitation and immunoblotting and in flow cytometry experiments where indicated. Anti-mouse siglec-1 Abs SER-4 and 3D6 were produced in-house (19, 20).

### Animals

Wild-type and siglec-E-deficient mice on C57BL/6J and Balb/c genetic backgrounds were generated as described previously (13, 21). Mice were bred and maintained under specific pathogen-free conditions within our own institutional colonies. WT and siglec-E-deficient mice were derived from heterozygous intercrosses and then maintained through homozygous crosses between WT mice and siglec-E-deficient mice. Periodically, the homozygous mouse colonies were refreshed by heterozygous intercrossing. Mice used in experiments were sex- and age-matched between the ages of 7 and 24 weeks. Animal experimentation was approved by the University of Dundee Animal Ethics Committee and carried out under UK Home Office Project License PPL60/3856.

### Immunofluorescence Staining

Cryostat sections of liver samples were prepared, fixed in pre-cooled 100% methanol at −20°C, and blocked with 10% normal serum (Gibco) in 1% fish skin gelatin prior to addition of primary Abs and secondary Abs. Tissue sections were counterstained with 400 ng/ml DAPI (Sigma), mounted in media (DAKOCytomation, USA) and analyzed by confocal microscopy. Excitation wavelengths were 405, 488, and 555 nm, and emission wavelengths maxima were 493/519 and 557/574 nm.

### Generation and Stimulation of BMDMs

Bone marrow cells were cultured in bacteriological plastic Petri dishes with DMEM media supplemented with penicillin and streptomycin, glutamine, 10% FBS, and either 20% L929 conditioned medium or M-CSF (25 ng/ml) for 7 days. BMDM were harvested with PBS supplemented with 3 mM EDTA and resuspended at a concentration of 1 × 10^6^/ml. For priming, 10 ml of BMDM cell suspension were seeded in 100 mm dishes and treated with 1 ng/ml LPS.

### Generation of BMDCs

Bone marrow cells were cultured in RPMI 1640 complete medium supplemented with penicillin and streptomycin, 10% FBS, 20 ng/ml recombinant mouse GM-CSF and 5 ng/ml IL-4 for 6 days or 10 ng/ml recombinant mouse GM-CSF and 1 ng/ml IL-4 for 12 days. The 12-day-cultured BMDC were stimulated for 24 h with 100 ng/ml LPS and analyzed by flow cytometry.

### Isolation of Peritoneal Macrophages

Cells were isolated from the peritoneal cavity by lavage with 5 ml RPMI. Cells were washed in media prior to plating in 24-well plates in RPMI containing 10% FBS, penicillin, and streptomycin. After 2 h, non-adherent cells were washed away and adherent macrophages were treated with LPS in complete RPMI media for 48 h.

### Flow Cytometry

Single cell suspensions were Fc-receptor-blocked for 30 min at 4°C with rat anti-mouse CD16/CD32 Ab in PBS with 1% FBS. Blocked cells were subsequently incubated with fluorophore-conjugated primary Abs for 60 min at 4°C, prior to washing in PBS containing 1% FBS and 2 mM EDTA. Following surface staining, cells were washed and analyzed by flow cytometry, or were fixed with 2% formaldehyde in PBS and then washed/permeabilized with BD perm/wash buffer (BD Biosciences), and stained with fluorophore-conjugated primary Abs for 60 min at 4°C. Cells were washed and intracellular fluorescence analyzed using a FACS Canto II flow cytometer and FlowJo software.

### Intracellular TNF-α Production

1 ng/ml LPS-primed cells were stimulated with 100 ng/ml LPS for 7 h and monensin-containing GolgiStop (BD Biosciences) was added in the last 6 h of culture. After washing, cells were surface stained with biotinylated sheep anti-siglec-E Ab followed by streptavidin-APC. Cells were then fixed with the Cytofix/Cytoperm solution (BD Biosciences) and incubated with PE-conjugated anti-mouse TNF-α Ab diluted in BD Perm/Wash buffer (BD Biosciences). Cells were analyzed by flow cytometry using a FACS Canto II with FlowJo software.

### Cytokine ELISAs

IL-6, RANTES, and IL-10 were measured in tissue culture supernatants and sera using ELISA kits according to the manufacturer’s instructions and assay procedures (Peprotech).

### Quantitative Real-time PCR

Total RNA was extracted using RNeasy mini kit (Qiagen). To quantify the gene expression, cDNA was synthesized using Omniscript RT kit (Qiagen). The sequences of the primers are shown below. Each PCR was performed in a 25 µl reaction mixture containing SYBR Green Universal master mix (Applied Biosystems). The final concentration of primers was 0.3 µM in each reaction. The thermal cycling conditions were as follows: 10 min at 95°C, followed by 40 cycles of 15 s at 95°C, 30 s at 60°C, and 30 s at 72°C.

**Table:** 

Siglec-E	Forward	GTC TCC ACA GAG CAG TGC AAC TTT ATC
	Reverse	TGG GAT TCA ACC AGG GGA TTC TGA G
Siglec-F	Forward	CCA CAG GAC CAC CCT CTC CTC
	Reverse	GGA CTT TAG TTC CTG TGT CAT CTC CC
Siglec-G	Forward	GCT GCT ACC TGA TAA AGA CAG TGC C
	Reverse	TTT CCA ATT CCG AGC CAG GGA CC
GAPDH	Forward	CAA CTC CCA CTC TTC CAC CTT CG
	Reverse	GTA GGG AGG GCT CAG TGT TGG G

### Treatment of Mice with LPS

Age- and sex-matched mice were injected intraperitoneally with 15 µg LPS [ultrapure *E. coli* 0111:B4 (Invivogen)]. After 3 h, mice were euthanized, blood was collected by cardiac puncture and serum samples were prepared for use in ELISA. In some experiments, livers and spleens were harvested and frozen for immunofluorescence staining and microscopy.

### Infection of Mice with *Salmonella*

Sex- and age-matched 9- to 15-week-old mice were infected by intravenous injection of *Salmonella enterica* serovar Typhimurium strain M525P suspensions in a volume of 0.2 ml PBS. Cultures were grown from single colonies in 10 ml LB broth incubated overnight without shaking at 37°C, then diluted in PBS to the appropriate concentration for inoculation. The infective dose was enumerated by plating dilutions onto LB agar plates. Mice were killed by exposure to a rising concentration of carbon dioxide, and death confirmed by cervical dislocation. Livers and spleens were aseptically removed and homogenized in sterile water using a Precellys 24 homogenizer. The resulting homogenate was diluted in a 10-fold series in PBS and LB agar pour plates were used to enumerate viable bacteria.

### Infection of Macrophages with Bacteria for Bacterial Uptake, Bactericidal Activity, and TLR4 Endocytosis Assays

To assess bacterial uptake, cells were infected with either *S*. Typhimurium strain M525P or *E. coli*-GFP for 30 min. After infection, the cells were washed with PBS and analyzed by flow cytometry. For assessing bactericidal activity, the infected cells were further incubated for 60 min with medium containing 100 µg/ml gentamicin to kill extracellular bacteria. The medium was then replaced with 10 µg/ml gentamicin and bactericidal activity was measured by harvesting cells at different time points and analyzing the decaying *E. coli*-GFP signal by flow cytometry. To assess TLR4 levels, cells were infected with *E. coli*-GFP for 1 h, stained with anti-TLR4 Ab and analyzed by flow cytometry.

### Siglec-E Co-Immunoprecipitation

Bone marrow-derived macrophages were primed with 1 ng/ml LPS for 3 days and lysed in 50 mM Tris–HCl, 150 mM NaCl, and 1% NP-40 with protease and phosphatase inhibitors. Lysates were subjected to immunoprecipitation with anti–siglec-E Ab. Immunoblots were probed with sheep anti-siglec-E Ab and Abs to SHP-1 and phosphotyrosine followed by HRP-conjugated secondary Abs followed by ECL autoradiography.

### SP3 Processing for Quantitative Proteomics

Bone marrow-derived macrophages were primed with 1 ng/ml LPS for 3 days and stimulated with 100 ng/ml LPS for 7 h. Monensin-containing GolgiStop (BD Biosciences) was added for the last 6 h of culture. After washing, cells were lysed in lysis buffer (4% SDS, 50 mM TEAB pH 8.5, 10 mM TCEP), boiled and sonicated with a BioRuptor (30 cycles: 30 s on, 30 s off) before alkylation with 20 mM iodoacetamide for 1 h at room temperature in the dark. Lysates were subjected to the SP3 protein clean-up procedure (22), eluted into digestion buffer (0.1% SDS, 50 mM TEAB pH 8.5, 1 mM CaCl2) and digested with trypsin at a 1:50 (enzyme:protein) ratio. TMT labeling and peptide clean-up were performed according to the SP3 protocol. Samples were eluted into 2% DMSO, combined, and dried under vacuum. TMT samples were fractionated using offline high pH reverse-phase chromatography. Peptides were separated, concatenated to 22 fractions, dried and peptides redissolved in 5% formic acid and analyzed by LC-MS.

### Proteomics Quantification and Bioinformatics Analysis

Four biological replicates from four independent biological samples were processed for proteomic analysis (22). The raw mass spectrometric data were loaded into MaxQuant (version 1.5.3.30) (23), using the Andromeda search engine software (24). Enzyme specificity was set to that of trypsin/P, allowing for cleavage of N-terminal to proline residues and between aspartic acid and proline residues. Other parameters used were as follows: (i) variable modifications—methionine oxidation, protein N-acetylation; (ii) fixed modifications, cysteine carbamidomethylation; (iii) database: Uniprot—mouse (downloaded 130501, 50800 sequences); (iv) labels: 10-plex TMT (v) MS/MS tolerance: FTMS- 50ppm, ITMS-0.5 Da; (vi) minimum peptide length, 7; (vii) maximum missed cleavages, 2; and (viii) and (ix) PSM and Protein false discovery rate, 1%. For bioinformatic analysis, Reporter ion intensities (corrected) results from MaxQuant were imported into Perseus software (version 1.5.1.6). The normalized corrected reporter ion intensities for each label were used to calculate ratios and all “Contaminant,” “Reverse” and “Only identified by site” proteins were removed from the data. Proteins above twofold change [log2(2) = 1], proteins with nominal *p*-value less than 0.05 [−log10(0.05) = 1.301] were considered as differentially expressed proteins. All bioinformatics analyses were performed with the Perseus software of the MaxQuant computational platform (23–25). GO over representation enrichment analysis was done using WEB-based Gene SeT AnaLysis Toolket and geneontology database (26). The mass spectrometry proteomics data have been deposited to the ProteomeXchange Consortium *via* the PRIDE (27) partner repository with the dataset identifier PXD008406.

### Statistics

Statistical significance was determined using the two-tailed Student’s *t*-test or non-parametric Mann–Whitney rank-sum test. All experiments were performed at least twice. *p* Values of <0.05 were considered significant.

## Results

### Siglec-E Is Upregulated on Macrophages by LPS *In Vivo* and *In Vitro*, but Does not Regulate Production of Inflammatory Mediators

To study the physiological role of siglec-E in regulating LPS-TLR4-driven inflammatory responses, we used siglec-E-deficient mice generated by three different approaches as described in our previous reports (13, 21). First, siglec-E KO1 mice (referred to as KO1) were generated in 129 embryonic stem (ES) cells following replacement of exons 1 and 2 with a neomycin cassette and backcrossed for more than 15 generations onto the C57BL/6J and Balb/c genetic backgrounds. Second, siglec-E “knockin” mice (referred to as R126D) were generated in C57BL/6 ES cells by introducing a targeted mutation, R126D, to destroy the sialic acid-binding site of siglec-E (13). R126D were shown previously not to express siglec-E protein at detectable levels due to effects on gene transcription (13). Third, siglec-E KO2 mice (referred as KO2) are a complete knock-out of siglec-E on a C57BL/6J background, generated by further crossing R126D mice with transgenic (Nes-cre)1Wme/J (Bal1 cre) mice to partially delete the loxP-flanked allele (21). As described previously, all mouse lines were born at normal Mendelian frequencies and were viable, with no alterations in leukocyte subpopulations compared with their matched WT controls.

**Figure 1:**
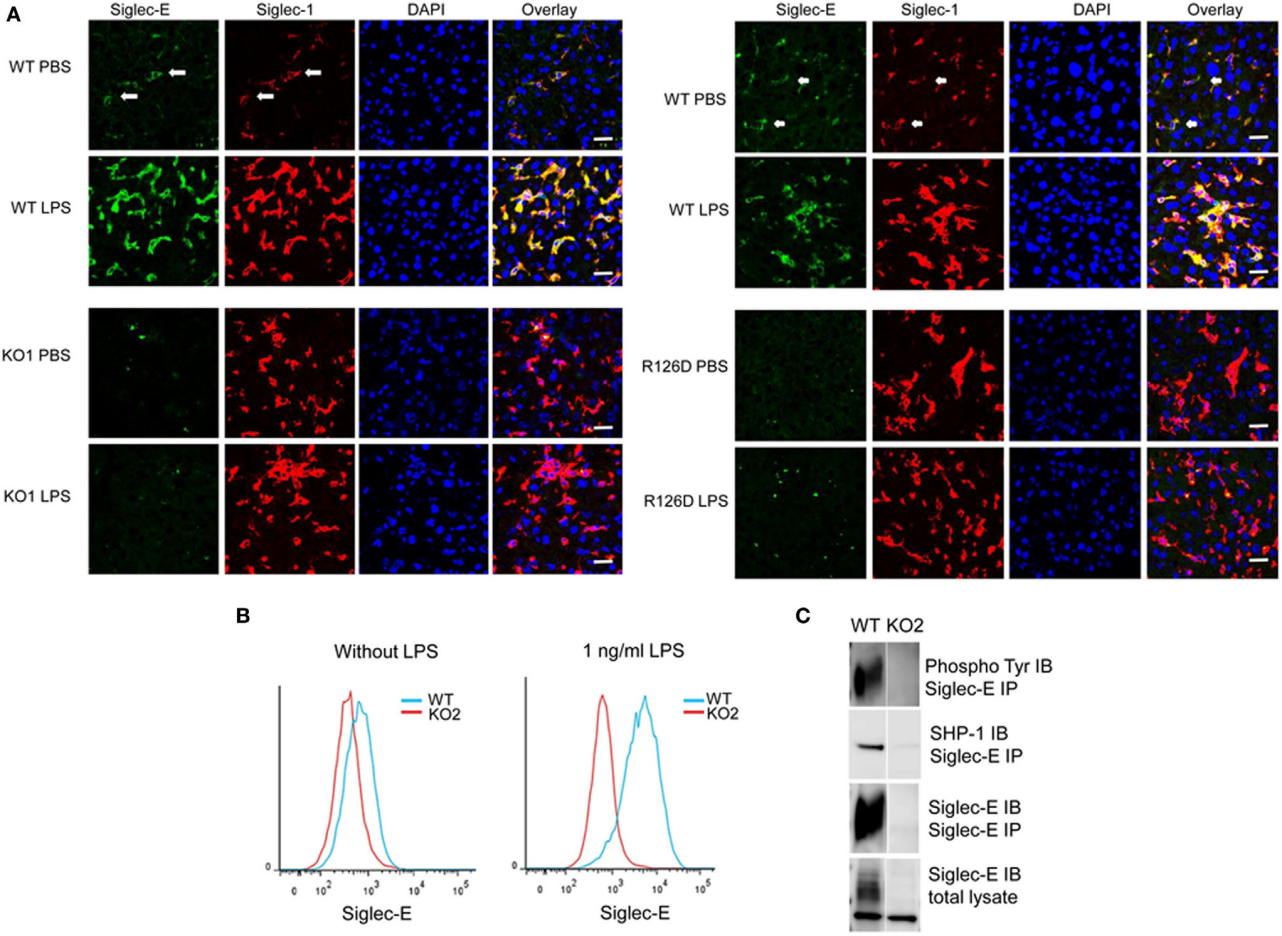
Siglec-E expression on tissue macrophages and bone marrow-derived macrophages (BMDM) is upregulated following lipopolysaccharide (LPS) treatment. **(A)** Wild-type (WT), KO1, and R126D mice were injected with 15 µg LPS or PBS intraperitoneally. After 3 h, animals were euthanized and tissue samples collected and frozen. Liver cryostat sections were labeled with sheep anti-siglec-E Ab directly labeled with Alexa 488 and anti-siglec-1 Abs SER-4 and 3D6 followed by anti-rat Alexa 647. Sections were also stained with DAPI to reveal nuclei. Siglec-E is expressed on Kupffer cells, which co-express siglec-1, and is upregulated following LPS stimulation in WT but not KO1 or R126D mice. Green dots in the anti-siglec-E stained KO1 and R126D sections are due to non-specific binding of the antibody (Ab). The scale bar represents 10 µm. **(B)** Siglec-E is expressed at low levels on BMDM and strongly upregulated following 3 days culture in 1 ng/ml LPS. **(C)** Siglec-E is constitutively phosphorylated in LPS-stimulated BMDM. WT and KO2 BMDM were treated for 3 days with 1 ng/ml LPS and the upregulated siglec-E was immunoprecipitated (IP) using sheep ant-siglec-E Ab and immunoblotted (IB) for phosphotyrosine, SHP-1 and siglec-E.

On cryostat sections, siglec-E was shown to be expressed in tissue macrophages including liver Kupffer cells (Figure 1A) and splenic red pulp macrophages (data not shown) and was strongly upregulated on Kupffer cells following injection of mice with 15 µg LPS (Figure 1). As expected, siglec-E was undetectable in tissues of siglec-E-deficient mice (Figure 1A). To study the signaling functions of siglec-E in macrophages, we used BMDM grown in M-CSF or in L929 cell conditioned medium as a source of M-CSF. These cells expressed very low levels of siglec-E but this could be strongly increased by cultivation for 3 days in low dose, 1 ng/ml LPS (Figure 1B). This low concentration of LPS was shown previously not to tolerize macrophages to a subsequent high-dose challenge of 100 ng/ml LPS (28). Immunoprecipitation of siglec-E from 1 ng/ml LPS-primed BMDM cells revealed that siglec-E was constitutively tyrosine-phosphorylated and associated with endogenous SHP-1 (Figure 1C).

**Figure 2:**
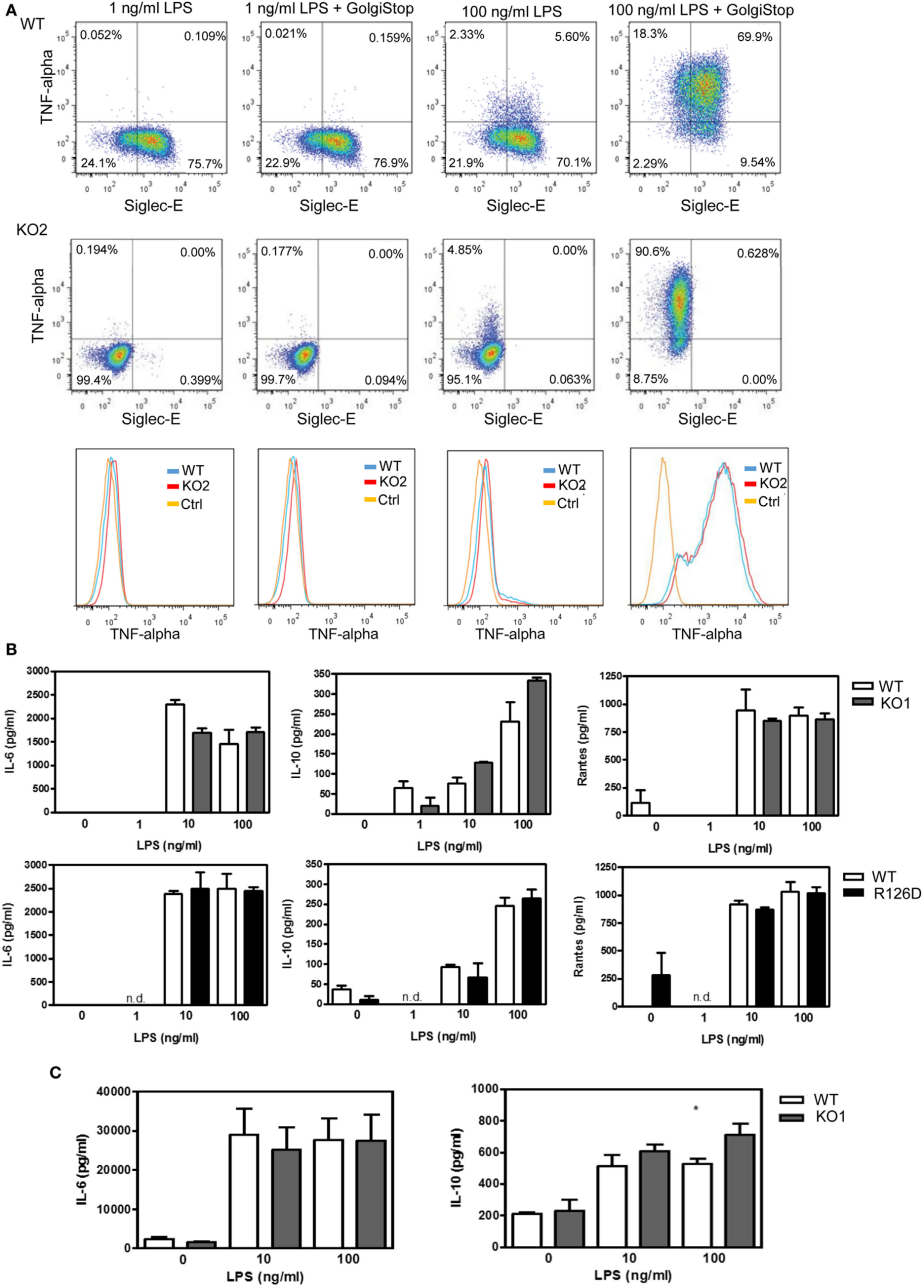
Analysis of cytokine production by wild-type (WT) and siglec-E-deficient macrophages following lipopolysaccharide (LPS) treatment. **(A)** WT and siglec-E KO2 bone marrow-derived macrophages (BMDM) were primed with 1 ng/ml LPS to induce siglec-E expression, treated with or without 100 ng/ml LPS ± GolgiStop to trap secretory proteins and analyzed by flow cytometry for TNFα. Three independent biological replicates were performed for each genotype. **(B)** BMDM from WT and siglec-E-deficient mice were cultured in the presence of LPS for 2 days. **(C)** Resident peritoneal macrophages from WT and siglec-E-deficient mice were cultured in the presence of LPS for 6 h. For **(B,C)**, cytokine levels in supernatants were assessed by ELISA. Data show means + 1 SD from a single experiment performed in triplicate and representative of two independent experiments. Statistical analyses were performed using Student’s *t*-test. *indicates *p* < 0.05.

**Figure 3:**
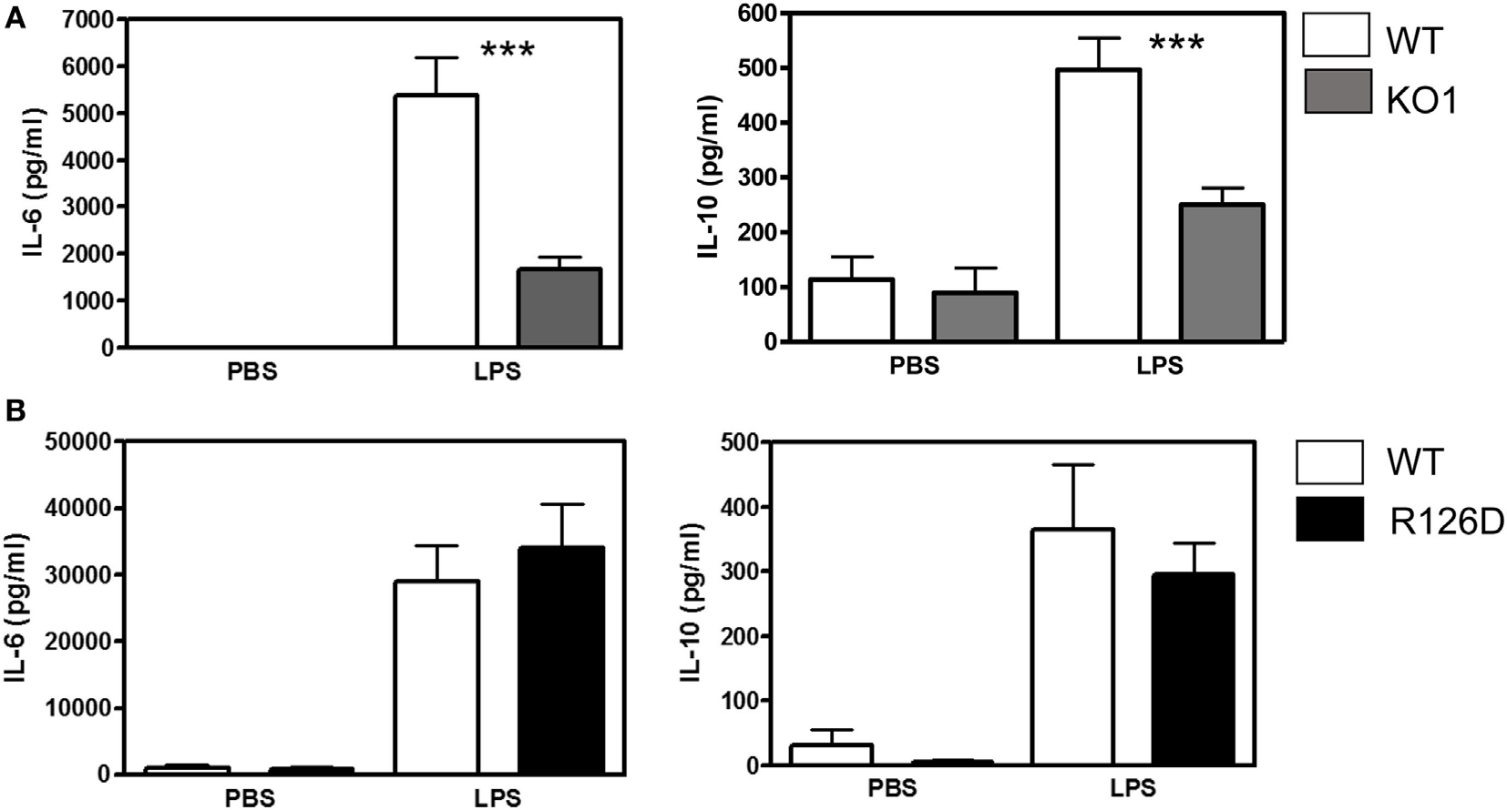
Analysis of lipopolysaccharide (LPS)-dependent cytokine responses in siglec-E-deficient mice. **(A)** Wild-type (WT) and KO1 mice and **(B)** WT and R126D mice were injected with 15 µg ultrapure LPS or PBS intraperitoneally and blood collected at 3 h. IL-6 and IL-10 concentrations in sera were measured by ELISA. Data show means + 1 SD from pooled samples, derived from two independent experiments, with three to six mice in each experiment. Statistical analysis was performed using Mann–Whitney *t*-test, ***indicates significant differences between WT and KO1 mice, *p* < 0.0005.

To investigate whether siglec-E could inhibit TLR4-driven inflammatory responses, LPS-primed BMDM were challenged for 7 h with 100 ng/ml LPS, with GolgiStop (monensin) added for the last 6 h post LPS stimulation to trap secreted inflammatory proteins. This led to strong induction of TNF-α as measured by intracellular flow cytometry (Figure 2A). However, no differences were seen comparing WT and siglec-E-deficient macrophages. To determine whether siglec-E could modulate other TLR4-driven cytokine responses at the later time point of 48-h post LPS treatment, ELISA was used to measure IL-6, IL-10, and RANTES in tissue culture supernatants, but no significant differences were seen comparing WT and siglec-E-deficient BMDM (Figure 2B). Similar observations were made using resident peritoneal macrophages that constitutively express siglec-E (Figure 2C). Finally, we asked whether siglec-E-deficient mice exhibited exaggerated cytokine responses at 3 h following intraperitoneal injection of LPS. Surprisingly, we saw reduced IL-6 and IL-10 responses in the sera of KO1 mice, but no differences were seen in E126D mice (Figure 3).

**Figure 4:**
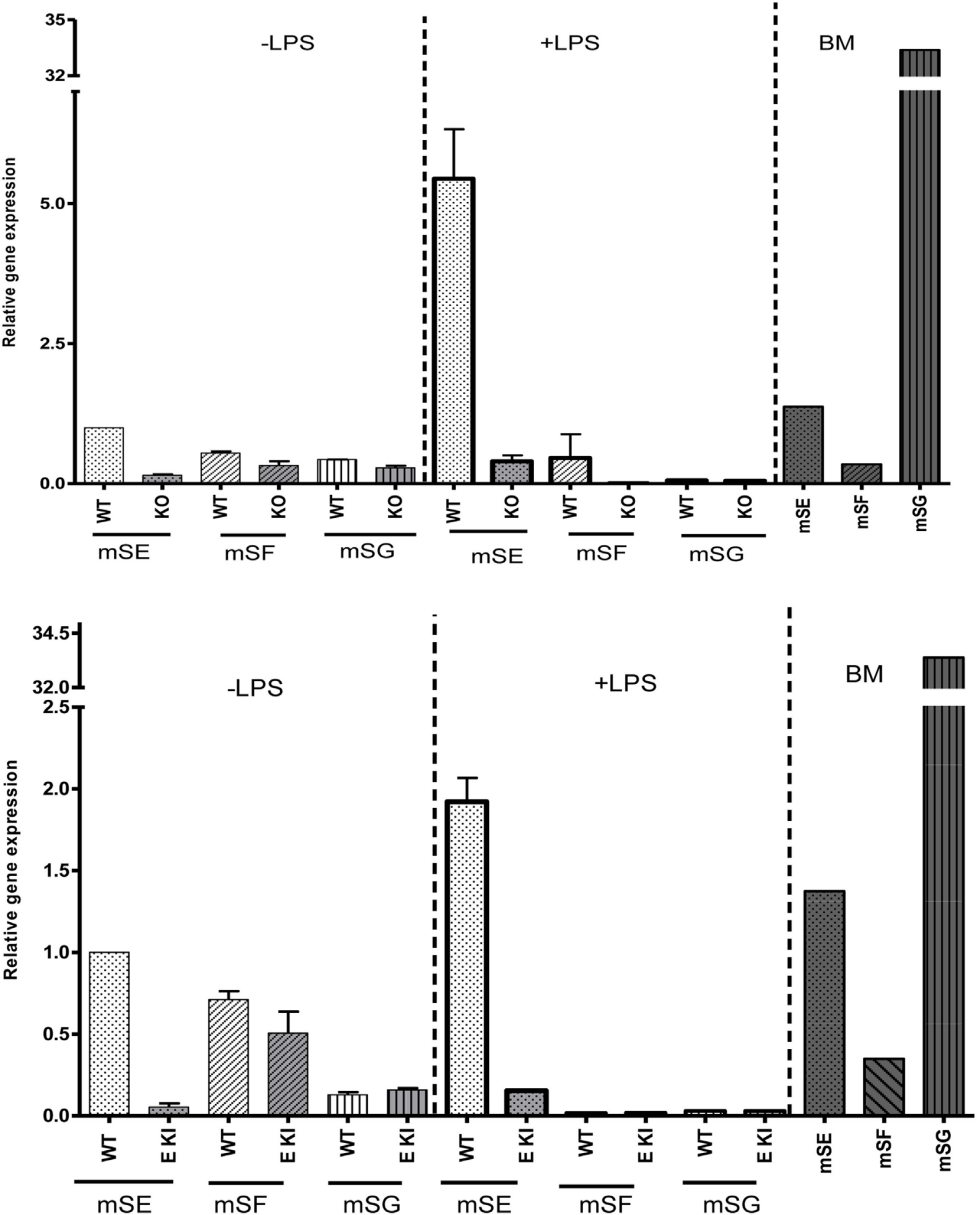
Other inhibitory CD33-related siglecs do not compensate for siglec-E deficiency in bone marrow-derived macrophages (BMDM). Relative gene expression of siglec-E, siglec-F, and siglec-G in BMDM untreated or stimulated with 10 ng/ml lipopolysaccharide (LPS) for 48 h from wild-type and siglec-E-deficient mice. Gene expression is shown relative to GAPDH, using untreated WT BMDM as calibrator. WT bone marrow cells were used as a control for siglecs-E, -F, and -G as they are expressed in developing neutrophils, eosinophils, and B cells, respectively, and show the expected gene expression profiles.

To check whether other ITIM-containing CD33-related siglecs were expressed in LPS-treated macrophages to compensate for the loss of siglec-E expression, we performed quantitative RT-PCR on macrophage lysates and analyzed expression of mRNAs encoding siglecs-E, -F, and -G, which are the only ITIM-bearing CD33-related siglecs in mice (Figure 4). As a positive control for siglecs-F and -G, which are mainly expressed in eosinophils and B cells, respectively, we used mouse bone marrow cells that showed the expected signals. However, while siglec-E mRNA was strongly upregulated in LPS-treated WT macrophages, there was no evidence for upregulation of mRNAs for siglecs-F and -G which remained at low or undetectable levels (Figure 4). Therefore, the failure of siglec-E to suppress TLR4 signaling cannot be explained by compensatory upregulation of other related inhibitory siglecs.

**Figure 5:**
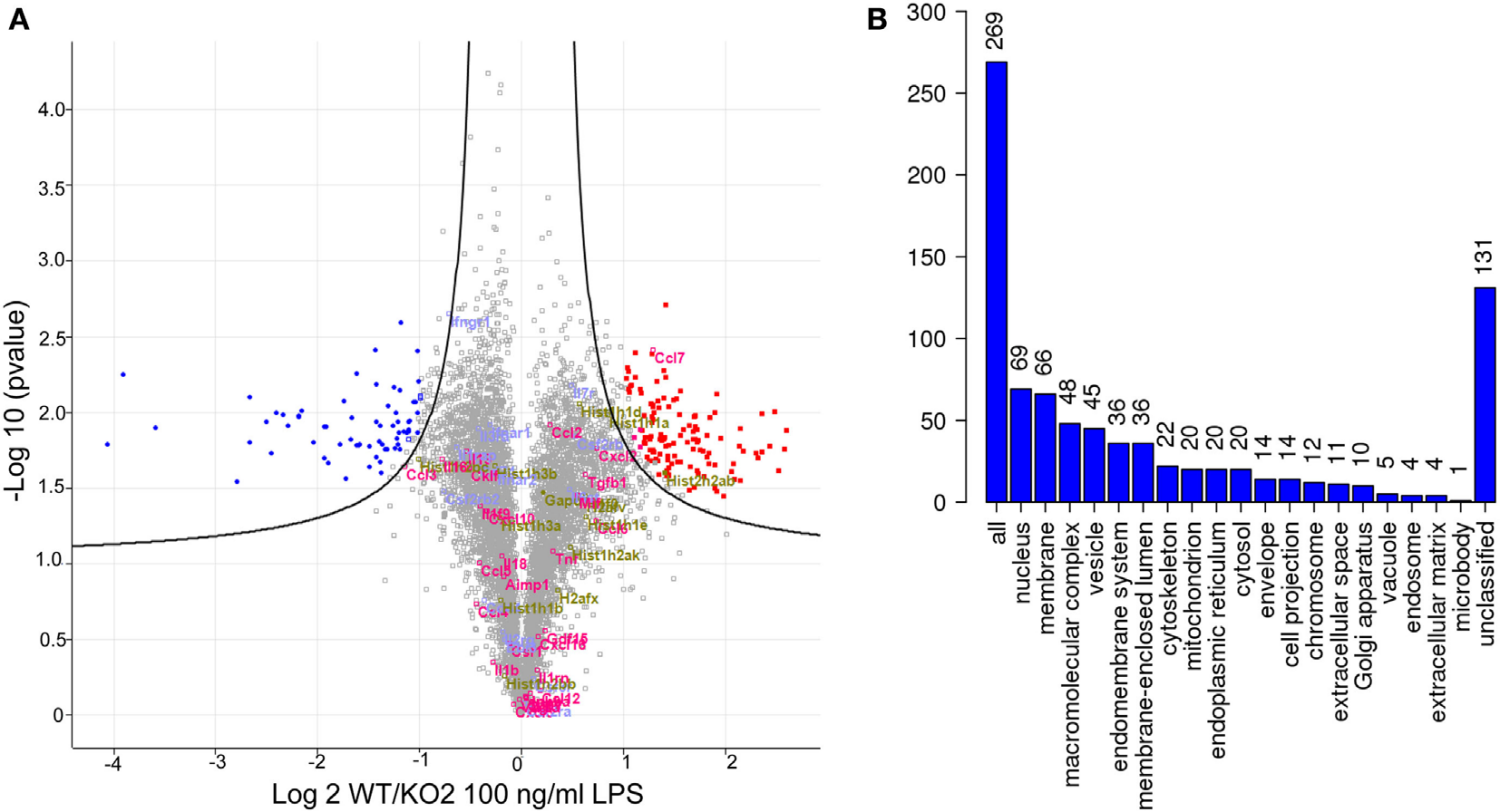
Quantitative proteomics analysis of wild-type (WT) and KO2 bone marrow-derived macrophages (BMDM) activated by lipopolysaccharide (LPS). **(A)** Volcano plot showing proteomics data derived from four independent biological replicates. WT and KO2 BMDM cells were primed for 3 days with 1 ng/ml LPS and challenged with 100 ng/ml LPS + GolgiStop for 7 h and lysates subjected to quantitative proteomics. The inflammatory mediators are annotated in pink. Gapdh and histone variants are in green and cytokine receptors in blue. **(B)** GO analysis of proteins that are differentially regulated between WT and KO2 BMDM.

Quantitative proteomics was next used as an unbiased approach to determine if additional LPS-induced inflammatory mediators could be regulated by expression of siglec-E in macrophages (Figure 5). A number of secretory inflammatory cytokines (TNF, Il16, Il1b, Il18, and Il19) and chemokines (Ccl2, Ccl3, Ccl4, Ccl5, Ccl6, Ccl7, Ccl9, Ccl12, Cxcl2, Cxcl3, Cxcl10, Cxcl16, and Cxxc1) were detected in the proteome following 100 ng/ml LPS treatment (Table S1 in Supplementary Material). However, apart from Ccl7 which was reduced in KO2 macrophages, none of the other upregulated cytokines and chemokines showed any significant differences comparing proteomes from WT and siglec-E-deficient BMDM (Figure 5A). Interestingly, the proteomics analysis revealed that WT and siglec-E-deficient BMDM exhibited many differentially regulated proteins not implicated in LPS-induced inflammation. Gene ontology enrichment analyses showed that these proteins were mainly associated with cellular features, such as membranes, vesicular transport, and cytoskeleton (Figure 5B).

### Siglec-E-Deficient BMDM Are Not Defective in Bacterial Uptake and Killing

**Figure 6:**
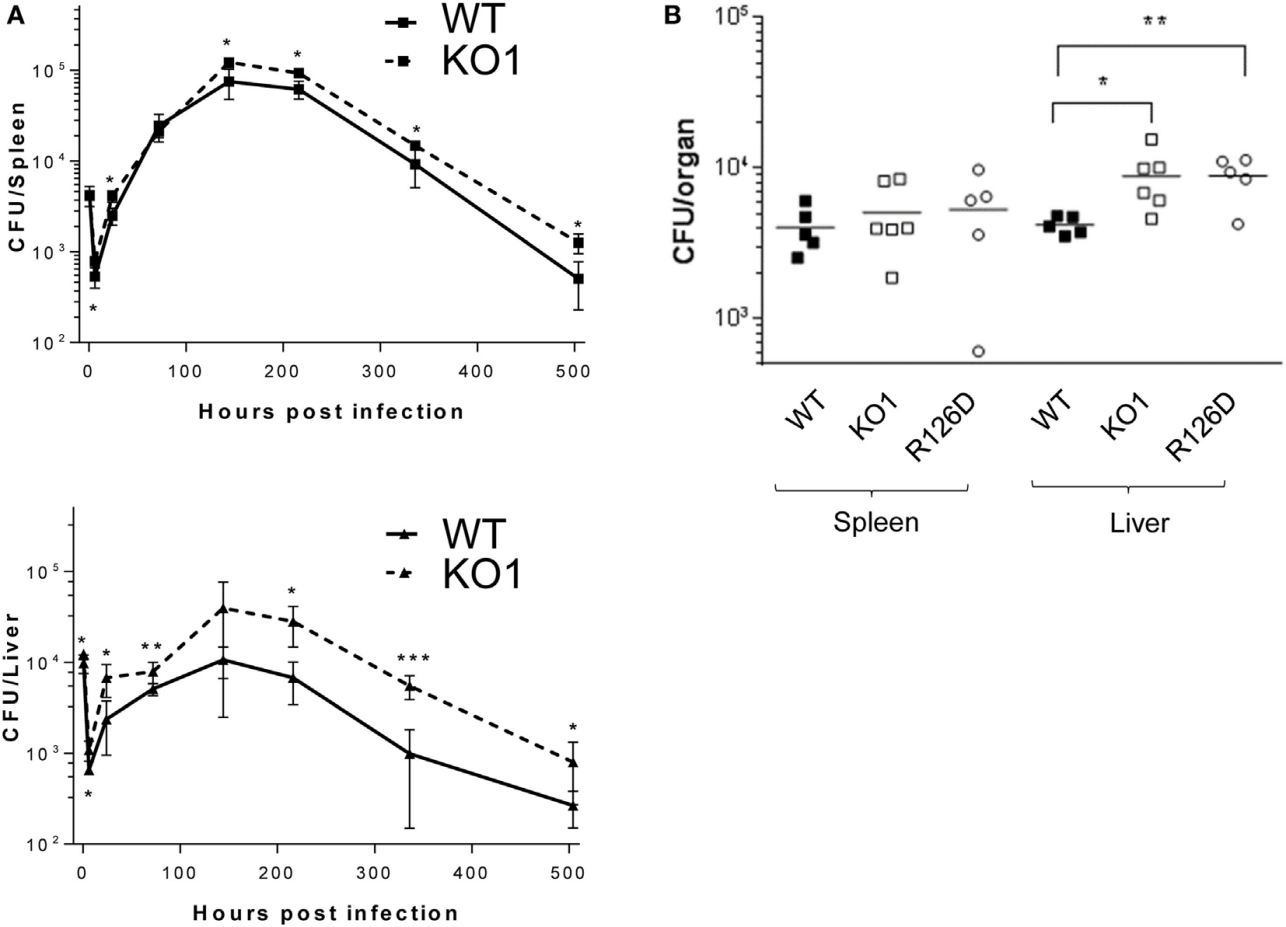
Siglec-E-deficient mice show enhanced growth of *Salmonella* Typhimurium following intravenous infection. **(A)** Wild-type (WT) and KO1 mice on a Balb/c background and **(B)** WT and KO1 on a C57BL/6J background and R126D mice were infected with *S*. Typhimurium M525P and liver and spleen CFU determined from groups of 4–8 mice at the indicated time points. Data in **(A)** show mean values ± 1 SD and data in **(B)** show values for individual mice. Statistical analysis was performed by Student’s *t*-test: *p < 0.05; **p < 0.005; ***p < 0.0005.

**Figure 7:**
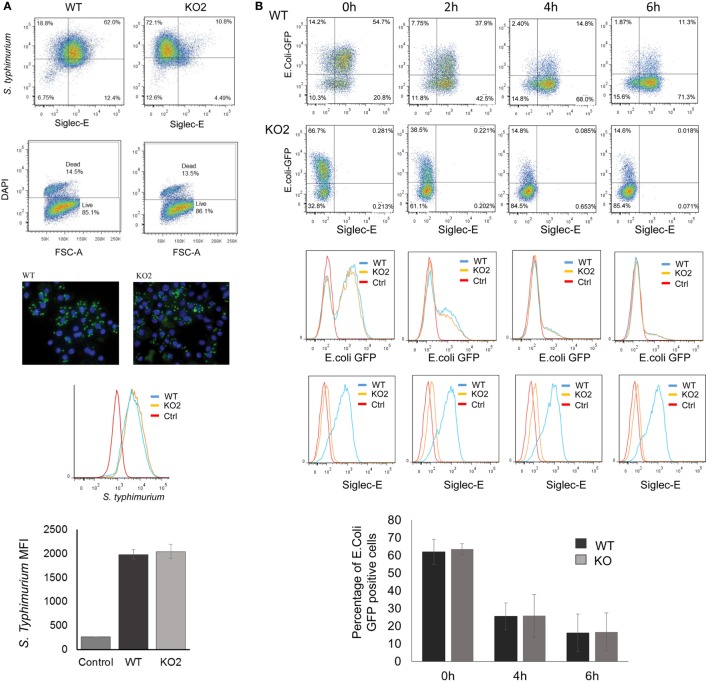
Uptake and killing of bacteria by bone marrow-derived macrophages (BMDM) are not affected by siglec-E expression. **(A)** BMDM from wild-type (WT) and siglec-E-deficient mice were cultured for 3 days with 1 ng/ml LPS to upregulate siglec-E expression and incubated with *S*. Typhimurium M525P for 30 min at a ratio of 1:50 BMDM:bacteria. Bacterial uptake was analyzed by flow cytometry using anti-salmonella antibody (Ab) conjugated with Alexa-488 and siglec-E expression was measured using biotinylated sheep anti-siglec-E Ab followed by streptavidin-APC. **(B)** BMDM as above were incubated with *E. coli*-GFP for 30 min at a ratio of 1:20 BMDM:bacteria. Cells were washed and maintained in antibiotic media to kill extracellular bacteria and the bactericidal activity of BMDM was monitored at the indicated time points by flow cytometry. No differences were seen in bacterial uptake or killing activity. Bar charts in **(A,B)** show mean values + 1 SD from three independent experiments.

The proteomic differences between WT and siglec-E KO macrophages point to functions relating to endocytosis and endosomal/lysosomal trafficking which could be relevant to bacterial uptake and/or bactericidal activity of macrophages (29–31). This possibility was also consistent with *in vivo* observations that siglec-E-deficient mice showed increased bacterial loads following infection with *Salmonella* Typhimurium (Figure 6). Therefore, to test the hypothesis that siglec-E contributed to uptake and killing of bacteria by macrophages, *in vitro* infection studies were carried out using *S*. Typhimurium and *E. coli* (Figure 7). No differences in uptake of either bacteria were observed at 30 min after infection comparing WT and siglec-E-deficient BMDM (Figure 7A). In addition, no differences in bactericidal activity of macrophages were seen using *E. coli*–GFP and measuring loss of the GFP signal over a 6 h time course (Figure 7B).

### Siglec-E Is Not Required for TLR4 Endocytosis in BMDM, BMDC, Splenic Macrophages, or Splenic DCs

**Figure 8:**
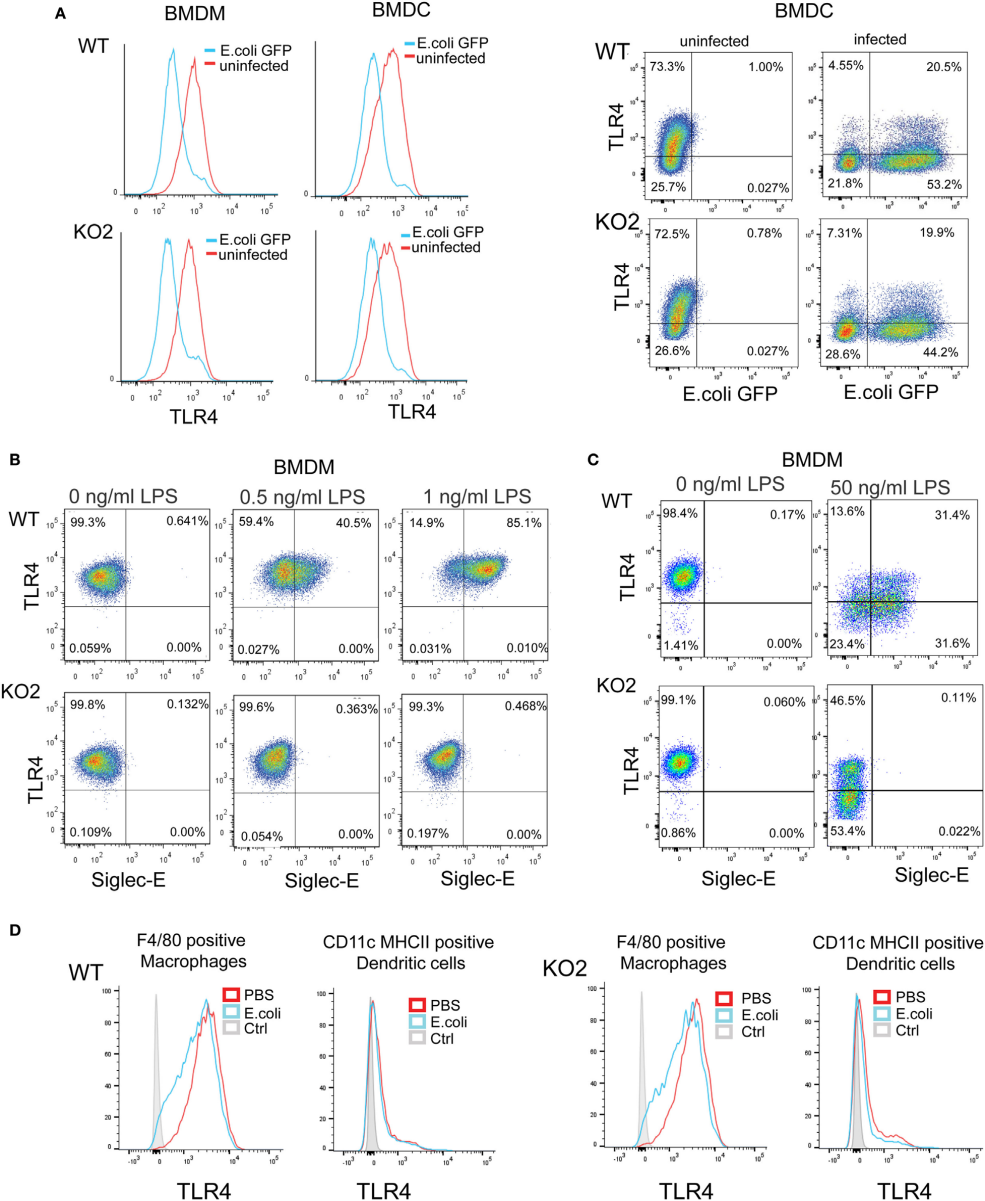
Expression of siglec-E does not influence toll-like receptor 4 (TLR4) endocytosis in bone marrow-derived macrophages (BMDM) and bone marrow-derived dendritic cells (BMDC). **(A)** Wild-type (WT) and siglec-E-deficient BMDM cultured for 3 days in 1 ng/ml lipopolysaccharide (LPS) and BMDC were incubated with *E. coli*-GFP for 1 h a ratio of 1:10 and TLR4 and GFP expression determined by flow cytometry **(B)** WT and siglec-E-deficient BMDM were incubated with the indicated doses of LPS for 3 days and TLR4 expression determined by flow cytometry. **(C)** BMDM were incubated for 1 day without LPS or with 50 ng/ml LPS and TLR-4 and siglec-E levels determined by flow cytometry **(D)** Splenocytes from WT and siglec-E-deficient mice were incubated with *E. coli*-GFP at 1:10 ratio and gated on F4/80 positive cells and CD11c, MHCII double-positive cells and expression of TLR4 determined by flow cytometry. Two independent biological replicates were performed for each genotype.

Several studies have shown that LPS–CD14–TLR4–MD2 complexes undergo endocytosis, leading to macrophage desensitization and tolerance (32–35). The endocytosed TLR4 initially activates TRIF–TRAM signaling in the early endosome and is later channeled to lysosomes and degraded through the ubiquitin pathway to limit further signaling (36–38). Recent studies in DCs demonstrated a role for siglec-E in promoting TLR4 endocytosis and downregulating TLR4-mediated inflammatory responses following *E. coli* infection (1, 18). In view of our findings that siglec-E on macrophages does not seem to regulate TLR4 inflammatory signaling, we asked if siglec-E affects TLR4 endocytosis in macrophages. Following *E. coli*-GFP infection of BMDM, TLR4 underwent endocytosis as reported by others (Figure 8A). However siglec-E-deficient BMDM showed similar levels of TLR4 endocytosis (Figure 8A; Figure S1A in Supplementary Material). To check if the previously reported role of siglec-E in downregulating TLR4 was restricted to DCs, we also analyzed responses in BMDC (Figure 8A). Similar to BMDM, the BMDC showed strong downregulation of TLR4 on exposure to *E. coli-*GFP, but this was unaffected in cells prepared from siglec-E deficient mice (Figure 8A). We also asked whether siglec-E might regulate LPS-mediated TLR4 endocytosis. While low doses up to 1.0 ng/ml LPS did not affect TLR4 levels at the cell surface, higher doses such as 50 ng/ml led to reduced TLR4 expression that was similar in WT and siglec-E-deficient BMDM (Figures 8B,C; Figure S1B in Supplementary Material). Similar observations were made with splenic macrophages and splenic DCs incubated with *E. coli*-GFP though the overall levels of TLR4 endocytosis were low compared to *in vitro* cultured BMDM and BMDC (Figure 8D; Figures S1C,D in Supplementary Material).

### Siglec-E Is Expressed by Splenic DC, Splenic Red Pulp Macrophages, and Splenic Granulocytes but Is Not Expressed on BMDCs

**Figure 9:**
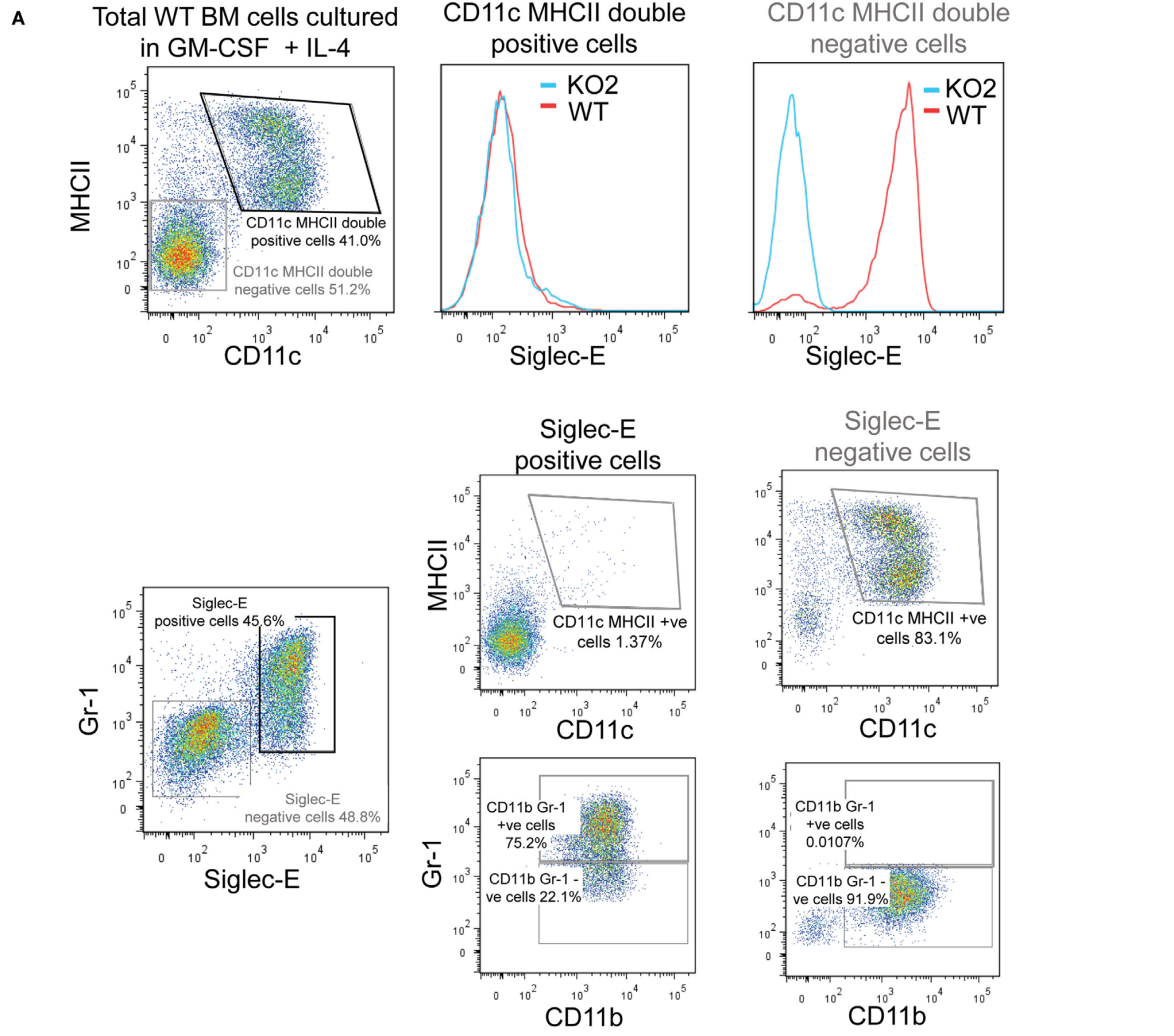

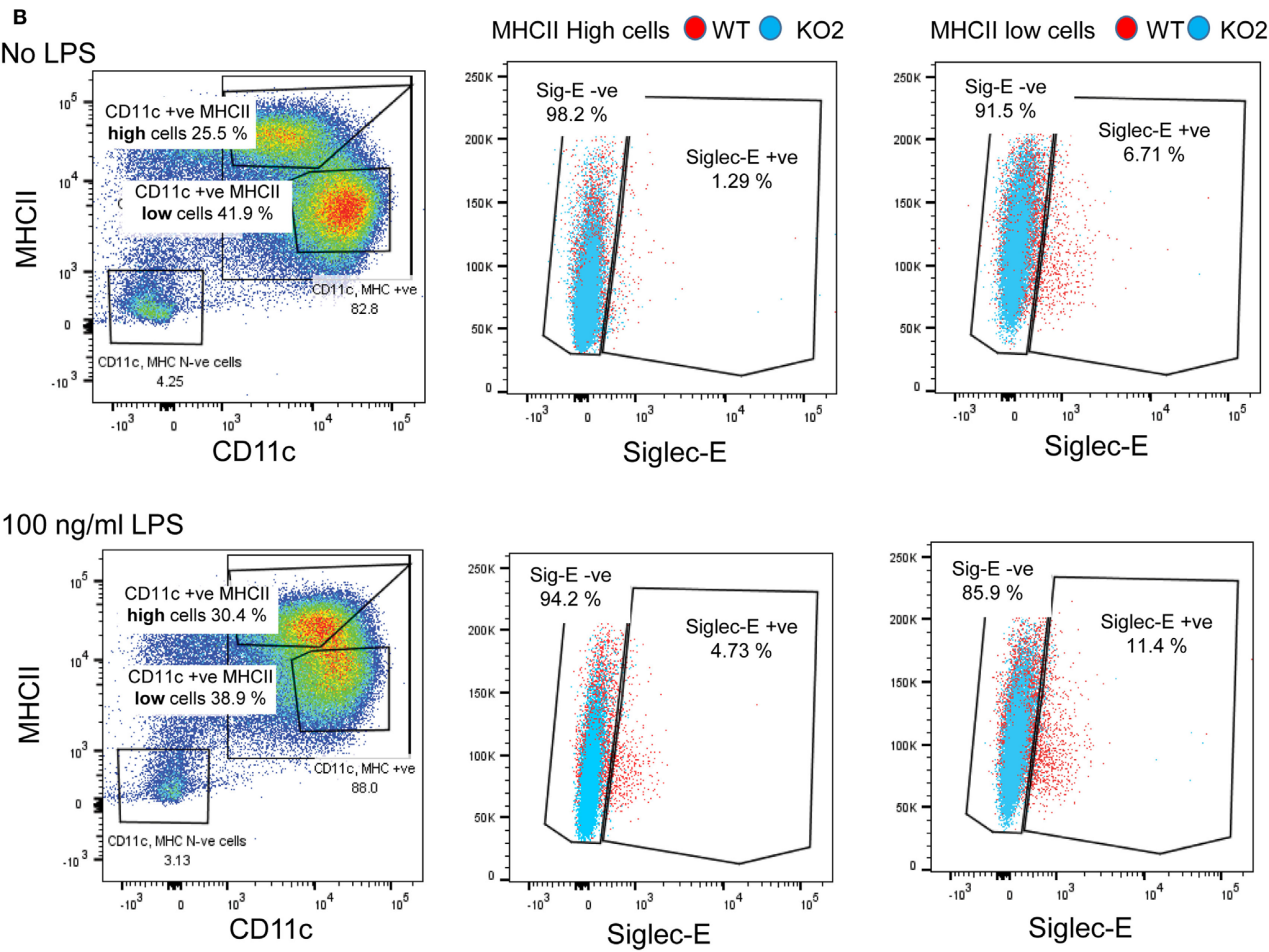
Siglec-E is not expressed by bone marrow-derived dendritic cells (BMDC). **(A)** Wild-type (WT) and siglec-E-deficient bone marrow cells were cultured in 20 ng/ml recombinant mouse GM-CSF and 5 ng/ml IL-4 for 6 days to generate BMDC and analyzed by flow cytometry for siglec-E expression on CD11c, MHCII double-positive cells and Gr-1 positive cells **(B)** BMDC were cultured in 10 ng/ml recombinant mouse GM-CSF and 1 ng/ml IL-4 and matured for 24 h using 100 ng/ml lipopolysaccharide (LPS) or left untreated and analyzed by flow cytometry. CD11c positive BMDCs with high and low levels of MHC expression were gated and siglec-E levels analyzed on the different subsets. Two independent biological replicates were performed for each genotype.

Previous studies have reported siglec-E-dependent functional responses using BMDC, but they did not demonstrate siglec-E expression by these cells (1, 18). To address this, we analyzed siglec-E levels in BMDC generated from BM progenitor cells cultured for 6 days in GM-CSF and IL-4 (Figure 9). As reported by others, the 6-day-cultured BM cells are a heterogeneous population containing granulocytes, macrophages, and DCs that can be readily distinguished using surface markers (39). In multiple experiments, we observed high siglec-E expression on Gr1-positive granulocytes, however, CD11c, MHCII double-positive DCs consistently lacked siglec-E expression (Figure 9A; Figure S1E in Supplementary Material). When 12-day-cultured BMDC were matured with LPS, there was clear upregulation of MHC class II, but more than 94% of the mature DCs (MHCII high cells) lacked siglec-E expression (Figure 9B).

**Figure 10:**
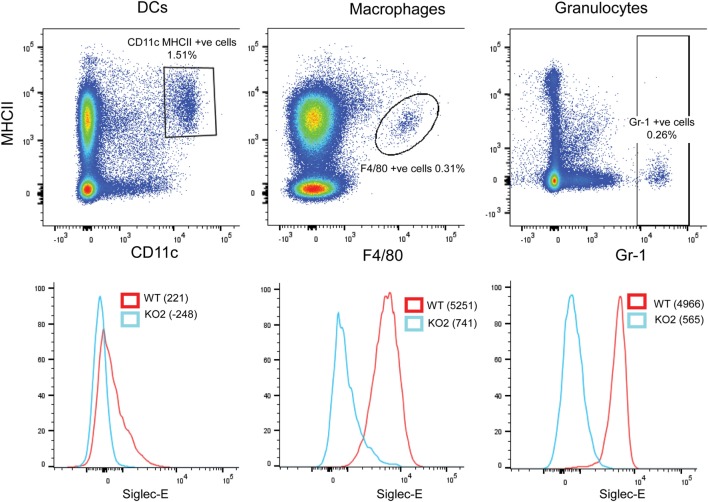
Differential expression of siglec-E on splenic myeloid cell populations. Wild-type (WT) and siglec-E-deficient splenocytes were gated for dendritic cells (DCs) (CD11c, MHCII double-positive cells), macrophages (F480 positive cells), and granulocytes (Gr-1-positive cells) and analyzed for siglec-E expression by flow cytometry. Two independent biological replicates were performed for each genotype.

In comparison to BMDC, CD11c, MHCII double-positive DCs extracted from the spleen expressed low levels of siglec-E as observed previously (13). By contrast, F4/80-positive splenic macrophages and Gr-1-positive granulocytes expressed much higher levels of siglec-E (Figure 10).

## Discussion

The focus of this study was to explore the role of siglec-E in regulating TLR4-induced inflammatory responses in macrophages. Our approach was to use siglec-E-deficient mice generated by gene targeting in ES cells, which is a conventional methodology to determine protein function in the immune system. Using three different strains of siglec-E-deficient mice and matched WT controls, we were unable to demonstrate an important role for siglec-E in regulating inflammatory cytokines downstream of TLR4 activation, both *in vitro* and *in vivo*. We showed that this was not due to compensation by other closely related inhibitory siglecs in the siglec-E-deficient macrophages. However, quantitative proteomics revealed differences in the levels of many proteins shared by WT and siglec-E-deficient macrophages following LPS activation and we cannot exclude the possibility that some of these differences could mask siglec-E dependent effects on TLR4 signaling.

In considering a potential role of siglec-E in regulating TLR4 signaling, it is important to distinguish *cis*-interactions of siglec-E with TLR4 (1, 18) and *trans*–interactions of siglec-E with other ligands. *Cis*-interactions occur through presentation of sialic acid ligands on neighboring glycoproteins and glycolipids. Their importance has been clearly documented in the case of CD22 and siglec-G on B cells using genetic and biochemical approaches where CD22 has been shown to associate with other CD22 molecules and siglec-G has been shown to associate with the B cell receptor complex [reviewed in Ref. (11)]. Such *cis*-interactions play a key role in regulating the threshold of B cell activation to antigens and preventing autoimmunity. The evidence that siglec-E associates *in cis* with TLR4 was based on pull-down experiments and overlays using recombinant forms of siglecs and TLRs, but no direct evidence that siglec-E associates with TLR4 *in situ* was provided (1). Experimentally induced *trans*-interactions of siglec-E occur following Ab cross-linking (14), exposure of macrophages to nanoparticles coated with sialic acid ligands (17), or to pathogens displaying a high density of sialic acids on their surface (16), leading to siglec-E-dependent suppression of TLR signaling. In these cases, the *trans*-ligand-induced clustering of siglec-E in the membrane is likely to drive strong ITIM phosphorylation and recruitment of effectors that could modulate TLR signaling through a number of pathways. Likewise, overexpression of siglecs in macrophage-like cell lines (15) could lead to non-physiological clustering and a similar outcome with respect to TLR-dependent signaling and suppression of pro-inflammatory cytokines. Based on the findings reported here, we propose that at physiological levels of siglec expression, stimulation of macrophages with LPS, in the absence of *trans*-ligand-induced siglec-E clustering, does not affect TLR4-dependent pro-inflammatory signaling. These observations are in line with a study showing that a sialic acid deletion mutant of Group B *Streptococcus* triggered similar amounts of TNF-α secretion in WT and siglec-E-deficient macrophages (16) and also with a study using lentiviral-mediated knockdown of siglec-E that did not affect the TLR4-triggered inflammatory response (17). In both cases, siglec-E inhibited LPS- or pathogen-induced inflammatory responses only *via trans*-interactions.

Our results contradict the findings of Chen et al. and Wu et al. who showed that BMDC from siglec-E-deficient mice exhibited strongly exaggerated IL-6 and TNF-α secretion following LPS and *E. coli* stimulation (1, 18). Furthermore, Wu et al. showed that TLR4 endocytosis induced by uptake of *E. coli* in DC populations was defective in cells prepared from siglec-E-deficient mice. This is at odds with findings presented here, where there was no difference in TLR4 endocytosis in both macrophages and DCs. Furthermore, we showed that siglec-E is not expressed on the vast majority of immature and mature BMDC, a key issue that was not addressed in their publications. Even splenic DCs expressed very low levels of siglec-E compared to macrophages and neutrophils, suggesting that siglec-E is not a major regulatory receptor in these cells. It is noteworthy that the siglec-E-deficient mice used in the studies of Chen et al. and Wu et al. were generated from 129 ES cells and backcrossed for three generations onto a C57BL/6 line resulting in less than 90% of the genome being derived from the C57BL/6 background. However, of more concern is the likely large number of “passenger genes” derived from 129 mice flanking the siglec-E locus that differ in protein sequence between siglec-E-deficient mice and the matched WT mice that could have strongly influenced the results of their studies. It is well established that many phenotypes ascribed to genes of interest are actually due to polymorphic passenger gene effects (40). This could also explain why the 129 ES-derived KO1 mice used in the present study showed reduced IL-6 and IL-10 responses to LPS *in vivo* whereas no differences were seen in C57BL/6 ES-derived R126D mice. Further studies are required to reconcile these differences.

The most interesting outcome of our unbiased quantitative proteomics was the finding that WT and siglec-E-deficient macrophages challenged with high-dose LPS exhibit differences in levels of many proteins associated with membrane function, vesicular transport, and cytoskeleton. Although we did not see altered bacterial uptake or killing *in vitro*, these protein differences might regulate aspects of macrophage function *in situ* that are important for host defense to infection and explain the reduced numbers of *Salmonella* seen here in WT mice compared to siglec-E-deficient mice. An additional attractive hypothesis is that siglec-E contributes to TLR4-induced macrophage differentiation and/or polarization. The heterogeneity of resident macrophage populations in different tissues is well documented, although the physiological relevance of this phenotypic heterogeneity within different tissue microenvironments is not completely understood (41). Several studies have shown that TLR agonists can drive polarization and cellular reprogramming in macrophages, monocytes, hematopoietic stem, and progenitor cells (42, 43). Induction of siglec-E by TLR4 activation could contribute to this process, as we showed that the induced siglec-E is constitutively tyrosine phosphorylated and associated with protein tyrosine phosphatase SHP-1. Interestingly, SHP-1 has been shown to be one of the key players among several molecular pathways that control macrophage polarization (44). Therefore, siglec-E-mediated activation of SHP-1 in macrophages could target key downstream substrates that modulate macrophage differentiation and TLR reprogramming, leading to a siglec-E-dependent phenotype. Our future studies will attempt to investigate the significance of this putative differentiation pathway for host defense and homeostasis.

## Ethics Statement

Animal experimentation was approved by the University of Dundee Animal Ethics Committee and carried out under UK Home Office Project License PPL60/3856.

## Author Contributions

MN designed the research, carried out experiments, analyzed results, and prepared the first draft of the manuscript. EM designed the research, carried out experiments, and analyzed results. HR designed the research, carried out experiments, and analyzed results. RS designed the research, carried out experiments, and analyzed results. ST analyzed results, and contributed to intepretation of data. PM designed experiments and interpreted results. PC led the research project, designed the experiments, interpreted the results, and wrote the final version of the manuscript.

## Conflict of Interest Statement

The authors declare that the research was conducted in the absence of any commercial or financial relationships that could be construed as a potential conflict of interest.

## Supplementary Material

Figure S1 Expression of siglec-E does not influence toll-like receptor 4 (TLR4) endocytosis in bone marrow-derived macrophages (BMDM) and bone marrow-derived dendritic cells (BMDC). **(A)** Wild-type (WT) and siglec-E-deficient BMDM were cultured for 3 days in 1 ng/ml lipopolysaccharide (LPS) and incubated with *E. coli*-GFP for 1 h at a ratio of 1:10 and TLR4 and GFP expression determined by flow cytometry. **(B)** BMDM were incubated for 1 day without LPS or with 50 ng/ml LPS and TLR4 levels determined by flow cytometry. **(C,D)** Splenocytes from WT and siglec-E-deficient mice were incubated with *E. coli*-GFP at 1:10 ratio and gated on F4/80 positive cells **(C)** and CD11c, MHCII double-positive cells **(D)** and TLR4 levels determined by flow cytometry. **(E)** Siglec-E is not expressed by BMDC. WT and siglec-E-deficient bone marrow cells were cultured in recombinant mouse GM-CSF and IL-4 for 6 days to generate BMDC and analyzed by flow cytometry for siglec-E expression on CD11c, MHCII double-positive cells. Staining for KO cells represents non-specific binding of the anti-siglec-E mAb. In **(A–E)**, data show means + 1 SD from two biological replicates.

TABLE S1 Quantitative proteomics data comparing WT and siglec-E-deficient macrophages stimulated with 100 ng/ml LPS. Data show log2 intensity values for 6,791 proteins identified in four independent biological replicates. Proinflammatory factors that are not significantly different comparing WT and siglec-E-deficient macrophages are highlighted in orange and proteins that are altered significantly are in green. Those increased in WT versus siglec-E-deficient macrophages are in dark blue, whereas those decreased in WT versus siglec-E-deficient macrophages are in pale blue.
