# Supplementary figures and images for: Expression of Siglec-E Alters the Proteome of Lipopolysaccharide (LPS)-Activated Macrophages but Does Not Affect LPS-Driven Cytokine Production or Toll-Like Receptor 4 Endocytosis
Source: Front Immunol. 2018 Jan 15;8:1926. doi: 10.3389/fimmu.2017.01926 (PMC5775731; doi:10.3389/fimmu.2017.01926)

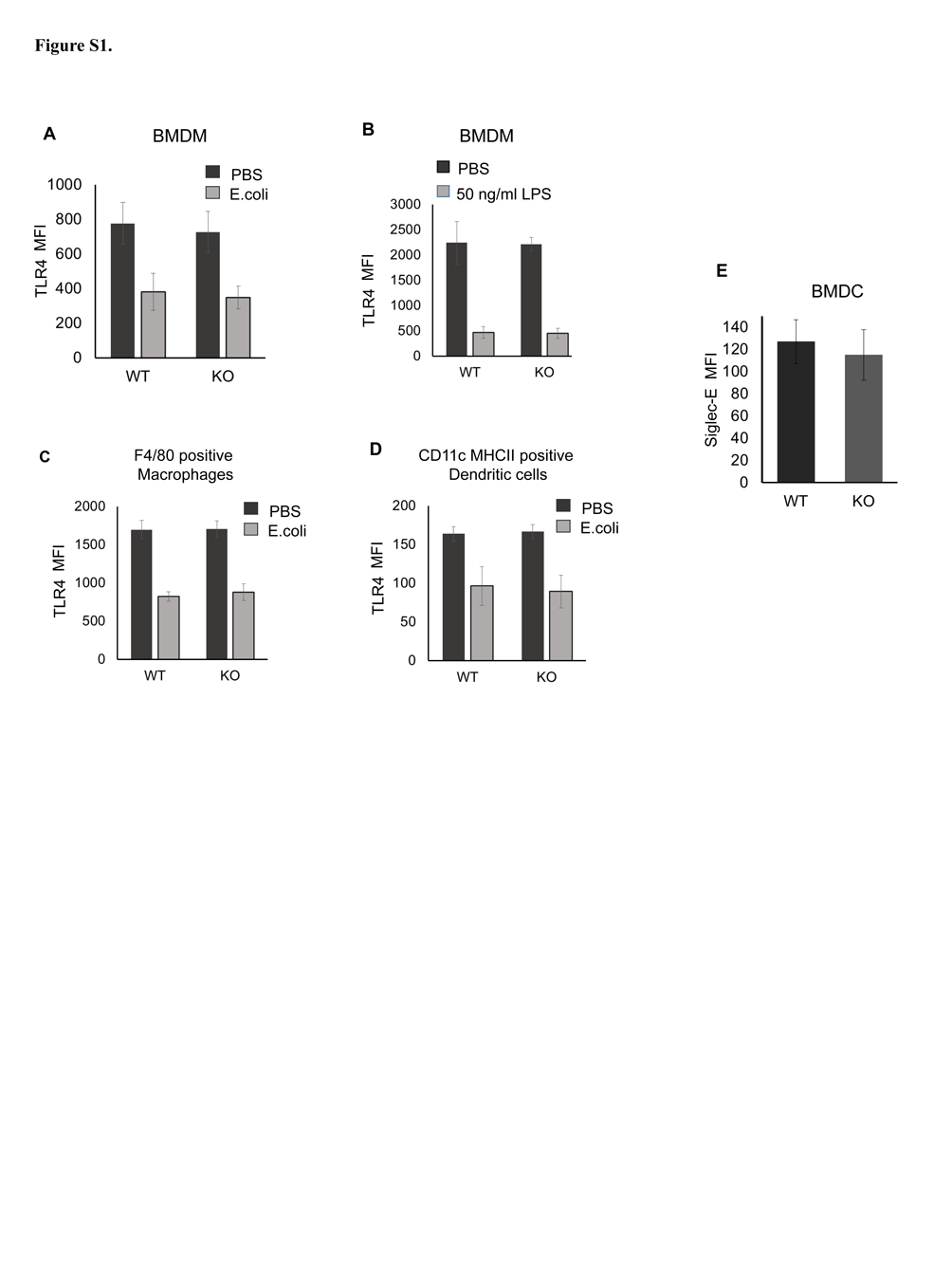

Supplement: Figure S1 — Expression of siglec-E does not influence toll-like receptor 4 (TLR4) endocytosis in bone marrow-derived macrophages (BMDM) and bone marrow-derived dendritic cells (BMDC). (A) Wild-type (WT) and siglec-E-deficient BMDM were cultured for 3 days in 1 ng/ml lipopolysaccharide (LPS) and incubated with E. coli-GFP for 1 h at a ratio of 1:10 and TLR4 and GFP expression determined by flow cytometry. (B) BMDM were incubated for 1 day without LPS or with 50 ng/ml LPS and TLR4 levels determined by flow cytometry. (C,D) Splenocytes from WT and siglec-E-deficient mice were incubated with E. coli-GFP at 1:10 ratio and gated on F4/80 positive cells (C) and CD11c, MHCII double-positive cells (D) and TLR4 levels determined by flow cytometry. (E) Siglec-E is not expressed by BMDC. WT and siglec-E-deficient bone marrow cells were cultured in recombinant mouse GM-CSF and IL-4 for 6 days to generate BMDC and analyzed by flow cytometry for siglec-E expression on CD11c, MHCII double-positive cells. Staining for KO cells represents non-specific binding of the anti-siglec-E mAb. In (A–E), data show means + 1 SD from two biological replicates. [file Image_1.tif]
